# c-Abl kinase regulates neutrophil extracellular trap formation and lung injury in abdominal sepsis

**DOI:** 10.1038/s41374-021-00683-6

**Published:** 2021-11-03

**Authors:** Avin Hawez, Zhiyi Ding, Dler Taha, Raed Madhi, Milladur Rahman, Henrik Thorlacius

**Affiliations:** Department of Clinical Sciences, Malmö, Section for Surgery, Skåne University Hospital, Lund University, 205 02 Malmö, Sweden

**Keywords:** Experimental models of disease, Inflammation

## Abstract

Sepsis is associated with exaggerated neutrophil responses although mechanisms remain elusive. The aim of this study was to investigate the role of c-Abelson (c-Abl) kinase in neutrophil extracellular trap (NET) formation and inflammation in septic lung injury. Abdominal sepsis was induced by cecal ligation and puncture (CLP). NETs were detected by electron microscopy in the lung and by confocal microscopy in vitro. Plasma levels of DNA-histone complexes, interleukin-6 (IL-6) and CXC chemokines were quantified. CLP-induced enhanced phosphorylation of c-Abl kinase in circulating neutrophils. Administration of the c-Abl kinase inhibitor GZD824 not only abolished activation of c-Abl kinase in neutrophils but also reduced NET formation in the lung and plasma levels of DNA-histone complexes in CLP mice. Moreover, inhibition of c-Abl kinase decreased CLP-induced lung edema and injury. Administration of GDZ824 reduced CLP-induced increases in the number of alveolar neutrophils. Inhibition of c-Abl kinase also markedly attenuated levels of CXC chemokines in the lung and plasma as well as IL-6 levels in the plasma of septic animals. Taken together, this study demonstrates that c-Abl kinase is a potent regulator of NET formation and we conclude that c-Abl kinase might be a useful target to ameliorate lung damage in abdominal sepsis.

## Abstract (web summary)

This study investigates the role of c-Abelson (c-Abl) kinase in neutrophil extracellular trap (NET) formation and inflammation during sepsis. The authors demonstrate that c-Abl kinase plays a pivotal role in NET formation, pulmonary formation of CXC chemokines and lung injury in sepsis. Authors conclude that targeting c-Abl kinase might be a useful strategy to ameliorate local and systemic inflammation in sepsis.

## Introduction

Abdominal sepsis is a life-threatening condition caused by an exaggerated and misdirected host responses characterized by wide-spread activation of innate immune cells^1^. Neutrophils are recognized to play a key role in sepsis^2,3^. On one hand, neutrophils are critical for eliminating invading microorganisms but on the other hand, excessive neutrophil responses cause organ damage and failure^4^. Convincing data have shown that neutrophil recruitment constitute a rate-limiting step in septic lung injury^2,5^. Beside secretion of antimicrobial compounds and phagocytic killing^6^, neutrophils can eliminate pathogens through expulsion of neutrophil extracellular traps (NETs) composed of neutrophil-derived DNA forming extracellular web-like structures decorated with nuclear histones as well as granular and cytoplasmic proteins^7,8^. NETs and their associated histones have been demonstrated to cause epithelial and endothelial cell damage^9^. Moreover, NETs provoke several pro-inflammatory effects in the blood^10^, pancreas^11^, liver^12^, and lung^10^. In fact, defective clearance of NETs increases organ damage in sepsis^13^. Although NETs seem to be important in the pathophysiology, the signaling mechanisms controlling NET generation in sepsis are not known.

Activation of neutrophils is mediated by multiple signaling pathways converging on specific transcription factors regulating adhesion and migration^2,14^. Many of these signaling cascades are controlled by intracellular kinases phosphorylating down-stream targets^15^. c-Abelson (c-Abl) kinase is a ubiquitously expressed non-receptor tyrosine-protein kinase, which was initially identified as a potent driver of myeloid cell transformation into leukemia^16^. More recent studies have shown that c-Abl kinase also plays a role in inflammation. For example, inhibition of c-Abl kinase protects against endotoxemic lung injury^17,18^, vascular leakage^19,20^, and IgG-induced glomerular injury^21^. In addition, it has been reported that c-Abl kinase regulates β_2_-integrin-mediated neutrophil migration via activation of Vav1^22,23^. Knowing that reactive oxygen species (ROS) are involved in the expulsion of NETs, it is interesting to note that c-Abl kinase has been implicated in the neutrophil formation of ROS^24^. Thus, accumulating data indicate that c-Abl kinase plays important functions in neutrophils and in several models of inflammation, which warrant further studies on the role of c-Abl kinase in NET formation and septic lung injury.

Based on these considerations, we hypothesized that c-Abl kinase activity might be involved in NET formation and lung damage in abdominal sepsis.

## Methods

### Animals

All experiments were conducted in accordance with the legislation on the protection of animals and approved by the Regional Ethical Committee for Animal Experimentation at Lund University, Sweden (Permit number: 5.8.18-08769/2019). Male C57BL/6 mice (20–25 g) were kept in a pathogen free facility on a 12–12 h light-dark cycle with free access to food and tap water. Mice were housed at least one week before use at a maximum of five mice per cage with environment enrichment. Animals were anesthetized with 75 mg of ketamine hydrochloride (Hoffman-La Roche, Basel, Switzerland) and 25 mg of xylazine (Janssen Pharmaceutica, Beerse, Belgium) per kg body weight. The ARRIVE guidelines were consulted for all animal experiments^25^.

### Experimental protocol of sepsis

Abdominal sepsis model was induced by cecal ligation and puncture (CLP) as previously described^26^. Briefly, animals were anesthetized and the abdominal wall was incised in the midline to mobilize the cecum. The cecum was filled with feces from ascending colon, ligated 75% of cecum (5-0 silk suture), soaked with PBS and punctured twice with a 21-gauge needle. The cecum was then returned to the peritoneal cavity and the abdominal incision was sutured. The specific c-ABL kinase inhibitor GZD824 (5 mg/kg, Selleck Chemicals, Houston, TX, USA) or vehicle (DMSO) was administered via the jugular vein 30 min prior to induction of abdominal sepsis. Sham mice underwent the identical laparotomy and resuscitation procedures, but the cecum was neither ligated nor punctured. Mice were then returned to their cages and reanesthetized for sample collection at 6 and 24 h after CLP induction. The left lung was ligated and excised for edema measurement. The right lung was used for collecting bronchoalveolar lavage fluid (BALF), in which neutrophils were quantified in a Burker chamber. Next, the lung was perfused with PBS, one part was fixed in formaldehyde for histology and the remaining lung tissue was snap-frozen in liquid nitrogen, and stored at −80 °C for later enzyme-linked immunosorbent assay (ELISA) as described subsequently.

### Isolation of blood neutrophils

Blood was collected from the inferior vena cava in acid citrate dextrose (1:10). Blood samples were added to Roswell Park Memorial Institute medium 1640 (RPMI 1640, Invitrogen, Stockholm, Sweden) supplemented with 10% fetal bovine serum (FBS, Invitrogen) and 2 mM EDTA (Sigma-Aldrich, Stockholm, Sweden). Following hypotonic lysis (5 ml ice-cold 0.2% NaCl, added for 45 s followed by addition of 5 ml 1.6% NaCl), neutrophils were separated from mononuclear cells by density gradient centrifugation using a Ficoll-Paque gradient (GE Healthcare, Uppsala, Sweden). The neutrophil layer was isolated and washed with RPMI 1640 and cells were resuspended at 4 × 10^6^ cells/ml. Next, the cells were homogenized and the activity of c-Abl kinase determined in isolated neutrophils as described below.

### Western blot

Isolated neutrophils were homogenized in ice-cold RIPA buffer (RIPA Lysis and Extraction Buffer, ThermoFisher, USA) containing protease inhibitors (Halt Protease Inhibitor Cocktail; Pierce Biotechnology, Rockford, IL) for 20 min and then sonicated and centrifuged (16,000 *g* for 15 min, 4 °C). Supernatants were collected and stored at −20 °C. Protein concentration of supernatants were determined by use of Pierce BCA Protein Assay Reagent (Pierce Biotechnology). Proteins (20 μg per lane) were separated by 8–16% Mini-PROTEAN^®^ TGX Stain-Free™ Gels (Bio-Rad) and transferred to polyvinylidene fluoride membranes (Novex, San Diego, CA, USA). Before blotting, total protein gel images were taken using Bio-Rad’s stain-free gel chemistry. Membranes were blocked in TBS/Tween 20 buffer containing 5% non-fat dry milk powder. Protein immunoblots were performed using rabbit anti-c-Abl kinase (2862, 1:1000, Cell signaling technology, Danvers, USA) and biotin-conjugated anti-phosphotyrosine antibody 4G10^®^ Platinum (16-452, 1:1000, Merckmillipore, Darmstadt, Germany) antibodies. Membranes were first incubated with HRP-conjugated anti-biotin secondary antibody (7075P5, 1:1000, Cell Signaling, Leiden, Netherlands) for detection of phosphor-c-Abl kinase. For detection of total c-Abl kinase same membranes were stripped off first and then incubated with anti-rabbit HRP-conjugated secondary antibody (7074, Cell Signaling, Leiden, Netherlands). Precision Plus Protein™ standards (1610363, BIO-RAD, Hercules, CA, USA) and Precision Protein™ StrepTactin-HRP conjugate (1610380, BIO-RAD) were used to determine the molecular weight of the c-Abl kinase. Protein bands were developed and analyzed using the BioRad ChemiDoc™ MP imaging system. Image Lab™ software version 5.2.1. was used to normalize target protein band signal against the total protein of respective lane before ratio calculation (Suppl. Fig. 1).

### Histology

Lung samples were fixed by immersion in 4% formaldehyde phosphate buffer overnight and then dehydrated and paraffin-embedded. Six µm sections were stained with haematoxylin and eosin. Lung injury was quantified in a blinded manner by adoption of a preexisting scoring system as previously described^27,28^ including size of alveolar spaces, thickness of alveolar septae, alveolar fibrin deposition and neutrophil infiltration graded on a zero (absent) to four (extensive) scale. Five random areas were scored and mean value was used. Histology score was the sum of all four parameters.

### Lung edema

Lung edema formation was measured using the lung wet-to-dry weight ratio. The left lung was excised, washed in PBS, gently dried using a blotting paper and weighed. The tissue was then dried at 60 °C for 72 h and re-weighed. The change in the ratio of wet weight to dry weight was used as indicator of lung edema formation.

### BALF

Animals were placed supine and the trachea was exposed by dissection. An angiocatheter was inserted into the trachea. BALF was collected by five washes of 1 ml ice-cold PBS containing 5 mM EDTA. The numbers of monomorphonuclear (MNL) and polymorphonuclear (PMNL) cells were counted in a Burker chamber.

### Systemic leukocyte count

Blood was collected from tail vein and mixed with Turks solution (0.2 mg gentian violet in 1 mL glacial acetic acid; 6.25% vol/vol) in a 1:20 dilution. Leukocytes were counted as MNL and PMNL leukocytes in a Burker chamber.

### ELISA

Plasma and lung levels of CXCL1, CXCL2 and IL-6 (R&D Systems, Abingdon, UK, Siemens, Marburg, Germany) were measured 24 h after induction of CLP. Linearity was assessed and confirmed by serial dilution of standards containing recombinant mouse CXCL1, CXCL2 and IL-6 in a calibrator diluent.

### Transmission electron microscope and scanning electron microscope

Deparaffinized lung tissue samples were fixed in 2.5% glutaraldehyde in 0.15 mol/L sodium cacodylate, pH7.4 (cacodylate buffer), for 30 min at room temperature. Specimens were washed with cacodylate buffer and dehydrated with an ascending ethanol series from 50% (vol/vol) to absolute ethanol (10 min/step). The specimens then were subjected to critical-point drying in carbon dioxide, with absolute ethanol as intermediate solvent, mounted on aluminum holders, and finally sputtered with 20 nm palladium/gold. Specimens were examined in a Jeol/FEI XL 30 FEG scanning electron microscope at the Core Facility for Integrated Microscopy at Panum Institute (University of Copenhagen, Denmark). The location of individual target molecules was analyzed at high resolution by ultrathin sectioning and transmission immunoelectron microscopy. Specimens on coverslips were embedded in Epon 812 and sectioned into 50-nm–thick ultrathin sections with a diamond knife in an ultramicrotome. For immunohistochemistry, sections were incubated overnight at 4 °C with primary antibodies against elastase (ab68672, 10 μg/ml, Abcam, Cambridge, UK) and citrullinated histone 3 (ab5103, 10 μg/ml, Abcam, Cambridge, UK). Controls without primary antibodies were included. The grids then were incubated with species-specific, gold-conjugated secondary antibodies (Electron Microscopy Sciences, Fort Washington, MD). Gold-labeled annexin V were also used. Finally, the sections were post-fixed in 2% glutaraldehyde and post-stained with 2% uranyl acetate and lead citrate. Specimens were observed in a Jeol/FEI CM100 transmission electron microscope operated at 80-kV accelerating voltage at the Core Facility for Integrated Microscopy at Panum Institute.

### DNA–histone complexes

Plasma levels of DNA–histone complexes were quantified by use of a sandwich ELISA based on monoclonal antibodies directed against histones and DNA according to the manufacturer’s instructions (Cell Death detection Elisa plus; Roche Diagnostics).

### NET formation in vitro

Bone marrow neutrophils (2 × 10^6^ cells/ml) were freshly isolated by density gradient centrifugation using a Ficoll-Paque gradient (GE Healthcare). Neutrophils were incubated with *100* *ng/ml tumor necrosis factor- α (TNF-α, 100* *ng/ml, Peprotech, London, UK) with and without GZD824 (500* *nM)* for 1 h at 37 °C. Samples were centrifuged (400 *g*, 5 min) and supernatants were collected to measure DNA-histone complex as described above. For detection of NETs by flow cytometry, neutrophils were first fixed with 2% formaldehyde and then washed two times with PBS containing 2% FBS. Cells were incubated with primary antibodies: PE-conjugated anti-Ly6G (551461, 5 μg/ml, clone 1A8, BD Pharmingen), fluorescein isothiocyanate conjugated anti-MPO (ab90812, 5 μg/ml, Abcam) and rabbit anti-H3cit (citrulline 2,8,17, ab5103, 5 μg/ml, Abcam, Cambridge, MA) antibodies in PBS containing 5% donkey serum. After washing two times, cells were incubated with rat anti-rabbit APC-conjugated secondary antibody (A-21038, 5 μg/ml, Thermo Scientific, Rockford, IL). For immunofluorescence imaging of NETs, neutrophils were stimulated by *TNF-α (100* *ng/ml) with and without GZD824 (500* *nM)* and stained on glass coverslips as described above. After immunostaining, coverslips were rinsed and mounted in fluoromount with *Hoechst 33342* (Thermo Fisher Scientific). Confocal microscopy was performed using LSM 800 confocal (Carl Zeiss, Jena, Germany) by a ×63 oil immersion objective (numeric aperture = 1.25). The pinhole was ~1 airy unit and the scanning frame was 1024 × 1024 pixels. Images were later processed using ZEN2012 software.

### ROS formation in neutrophils

Bone marrow neutrophils were isolated as described above. Neutrophils were incubated with anti-CD16/CD32 (553142, 5 μg/ml, BD Pharmingen) to block Fc_ϒ_III/IIRs and reduce non-specific labeling and APC-conjugated anti-Ly6G (560599, 5 μg/ml, clone 1A8, BD Pharmingen, San Jose, CA) antibodies. To detect ROS (superoxide and hydrogen peroxide) generation, cells were incubated with 1 μM dihydroethidium (D23107, Thermo Fisher Scientific) for 15 min at 37 °C and then stimulated with 100 ng/ml TNF-α for 20 min at 37 °C with and without GZD824 (500 nM). Flow cytometry analysis was performed on a CytoFLEX flow cytometer (Becton Dickinson, Mountain View, CA, USA), and viable gate was used to exclude dead and fragmented cells.

### Statistics

Data are presented as mean values ± standard error of the mean. For two group comparisons, Mann-Whitney rank sum test was used. *P* < 0.05 was considered statistically significant and *n* represents the number of animals or experiments.

## Results

### c-Abl kinase activity in neutrophils

**Fig. 1:**
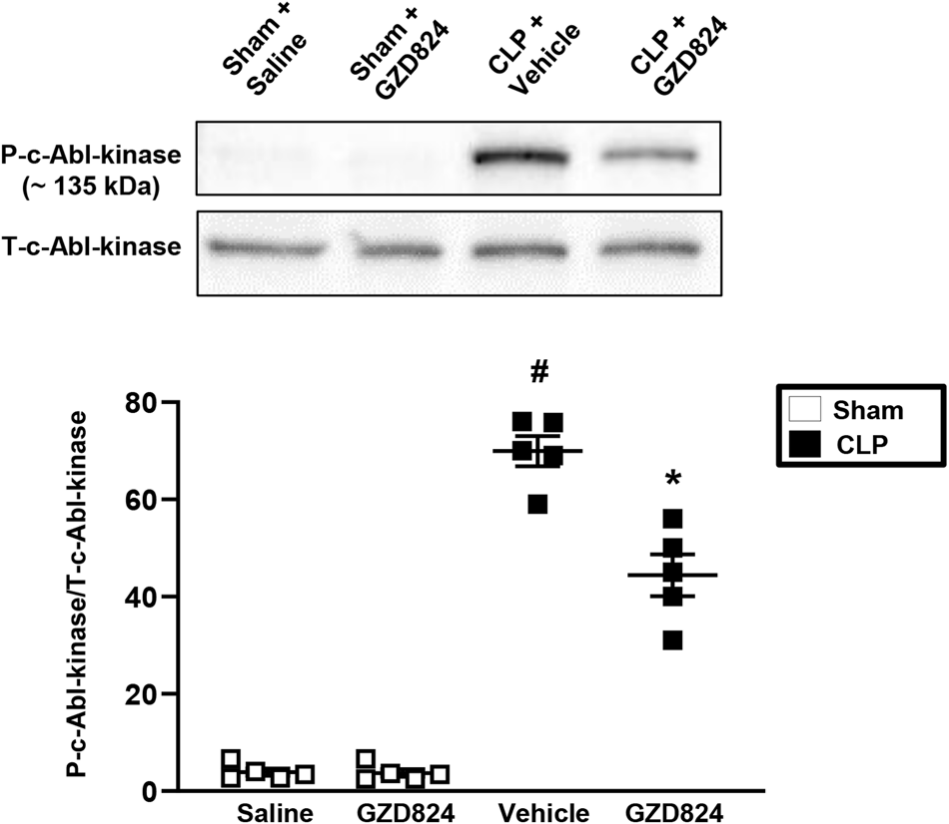
c-Abl kinase activity in neutrophils. Phosphorylation of c-Abl kinase in circulating neutrophils were examined by western blot as described in Materials and Methods. Animals were treated with GZD824 (5 mg/kg) or vehicle prior to CLP induction. Mice treated with saline (sham) or GZD824 alone without CLP. Samples were collected 24 h after induction of CLP. Data are presented as mean values ± standard error of the mean (SEM) and *n* = 5. ^#^*P* < 0.05 vs. Sham and *P < 0.05 *vs*. Vehicle + CLP.

To determine systemic activation of c-Abl kinase in sepsis, western blot was used to analyze c-Abl phosphorylation in circulating neutrophils from CLP mice. It was found that CLP enhanced phosphorylation of c-Abl kinase in circulating neutrophils (Fig. 1). In addition, treatment with GZD824 markedly decreased c-Abl kinase activation in neutrophils, indicating that GZD824 is an effective inhibitor of c-Abl kinase activity in sepsis (Fig. 1). Injection of GZD824 alone had no effect on c-Abl kinase phosphorylation (Fig. 1).

### c-Abl-kinase regulates NETs formation in sepsis

**Fig. 2:**
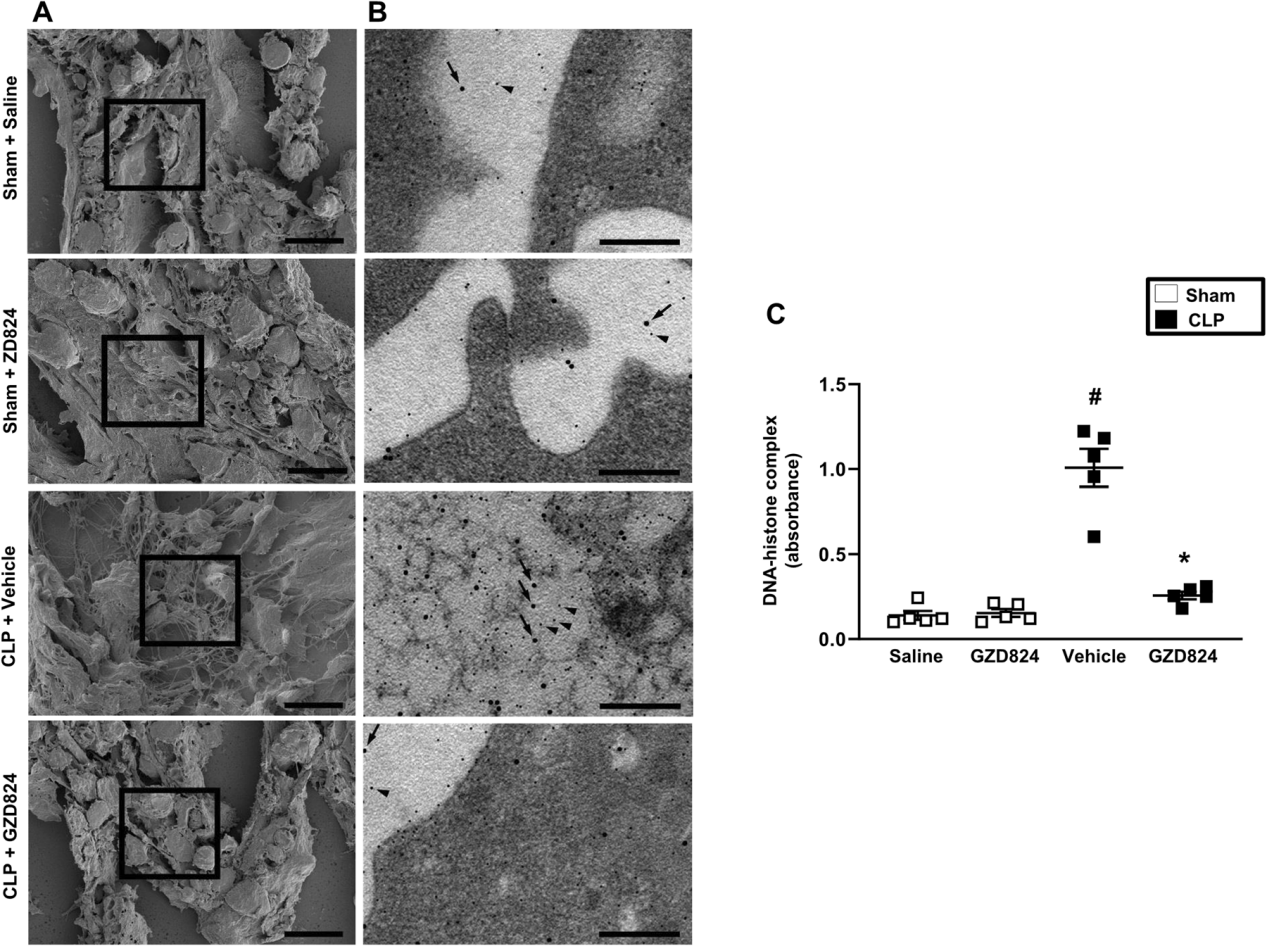
NET formation in sepsis. **A** Scanning electron microscopy showing extracellular web-like structures in the lung from CLP mice. Scale bar = 5 μm. **B** Transmission electron microscopy of the indicated area of interest from Fig. 2A incubated with gold-labeled antibody against citrullinated histone 3 (large gold particles) and anti-elastase (small gold particles) antibodies. Scale bar = 0.25 μm. All images are representative of five independent experiments. **C** DNA–histone complex formation. Animals were treated with GZD824 (5 mg/kg) or vehicle prior to CLP induction. Mice treated with saline (sham) or GZD824 alone without CLP. Samples were collected 24 h after induction of CLP. Data are presented as mean values ± standard error of the mean (SEM) and *n* = 5. ^#^*P* < 0.05 *vs*. Sham and *P < 0.05 vs. Vehicle + CLP.

**Fig. 3:**
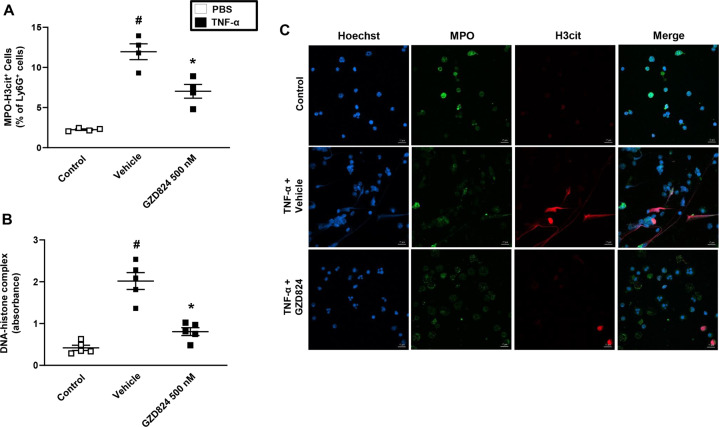
NET formation in neutrophils. NETs were generated from isolated neutrophils by TNF-α-stimulation, co-incubated with or without GZD824 (500 nM). Non-stimulated neutrophils served as a control. **A** Levels of citrullinated histone 3 and MPO in isolated neutrophils detected by flow cytometry and **B** DNA-histone complexes in the supernatant determined by ELISA. Data represent means ± SEM and *n* = 5. ^#^*P* < 0.05 versus control mice and *P < 0.05 versus Vehicle + TNF-α. **C** Neutrophils were immune-stained with antibodies to citrullinated histone 3 (H3cit), myeloperoxidase (MPO), and DAPI nuclear stain. Representative confocal fluorescence microscopy images from four independent experiments. Scale bar indicates 10 μm.

**Fig. 4:**
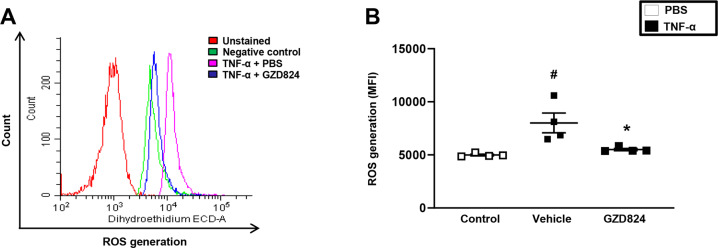
ROS formation in neutrophils. Quantification of ROS formation in isolated neutrophils by flow cytometry. Neutrophils were stimulated by TNF-α with or without GZD824 (500 nM). Nonstimulated neutrophils served as a control. **A** Representative histogram of ROS generation and **B** aggregate data. Data are presented as mean values ± standard error of the mean (SEM) and *n* = 5. ^#^*P* < 0.05 versus control mice and *P < 0.05 versus Vehicle + TNF-α.

By use of scanning electron microscopy, we observed that CLP induced formation of extracellular fibrillar and web-like structures in the lung compatible with NETs (Fig. 2A). In addition, transmission immunoelectron microscopy revealed that the neutrophil-derived granule protein elastase and citrillunated histone 3 co-localized with these extracellular fibrillar and web-like structures (Fig. 2B), which were not observed in the normal lung. It was observed that administration of GZD824 greatly reduced generation of NETs in the septic lung (Fig. 2B). Moreover, plasma levels of DNA-histone complexes were increased by eightfold in CLP animals (Fig. 2C). Treatment with GZD824 decreased CLP-induced increases of DNA–histone complexes in plasma by 83% (Fig. 2C). Administration of GZD824 alone had no effect on NET formation in mice (Fig. 2A–C). To further study the role of c-Abl kinase in regulating NET formation in neutrophils, we used isolated bone marrow neutrophils in vitro. Stimulation of neutrophils with TNF-α markedly increased neutrophil co-expression of neutrophil-derived granule protein MPO and citrullinated histone 3 (Fig. 3A) as well as DNA–histone complexes (Fig. 3B). Indeed, co-incubation of neutrophils with GZD824 dose-dependently decreased TNF-α-induced co-expression of MPO and citrullinated histone 3 (Fig. 3A) levels as well as DNA–histone complexes (Fig. 3B) in isolated neutrophils, demonstrating that c-Abl kinase regulates NET formation in neutrophils. In addition, using confocal fluorescence microscopy, we found that TNF-α challenge induced formation of DNA fibrillar and web-like structures that co-localized with MPO and citrullinated histone 3 (Fig. 3C). Co-incubation of neutrophils with GZD824 prevented TNF-α-provoked induction of these DNA structures containing MPO and citrullinated histone 3 (Fig. 3C), suggesting that c-Abl kinase controls NET formation. Knowing that ROS plays a key role in NET formation^29^, it was of interest to study the role of c-Abl kinase in TNF-α-induced formation of ROS. TNF-α stimulation triggered clear-cut formation of ROS in isolated neutrophils (Fig. 4A, B). Co-incubation of neutrophils with GZD824 markedly decreased TNF-α-provoked generation of ROS in neutrophils (Fig. 4A, B).

### c-Abl kinase mediates septic lung injury

**Fig. 5:**
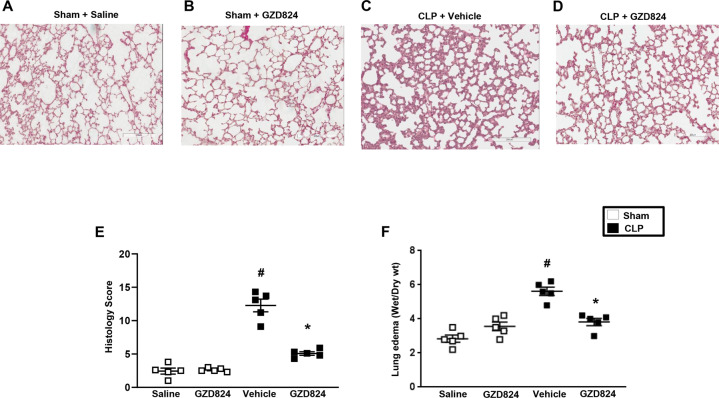
Representative haematoxylin & eosin sections of the lung. **A** Animals were treated with saline or **B** GZD824 alone. Separate mice were pretreated with **C** vehicle or **D** 5 mg/kg of GZD824 prior to CLP induction. **E** Lung injury score and **F** edema formation were quantified as described in Materials and Methods. Data are presented as mean values ± standard error of the mean (SEM) and *n* = 5. ^#^*P* < 0.05 vs. Sham and *P < 0.05 *vs*. Vehicle + CLP. Samples were harvested 24 h after CLP induction. Scale bar indicates 100 μm.

Histological analysis showed normal lung architecture in sham mice (Fig. 5A). CLP caused severe pulmonary injury, characterized by destruction of tissue microarchitecture, edema of interstitial tissue, and infiltration of neutrophils (Fig. 5C). Administration of GZD824 decreased CLP-induced tissue destruction and neutrophil infiltration in the lung (Fig. 5D). Quantification revealed that CLP-evoked lung damage was reduced in GZD824-treated animals (Fig. 5E). CLP caused clear-cut edema formation in the lung, reflected by an enhanced lung wet-to-dry ratio (Fig. 5F) Treatment with GZD824 decreased lung wet-to-dry ratio by 89% in septic animals (Fig. 5F).

### c-Abl kinase regulates neutrophil recruitment

**Fig. 6:**
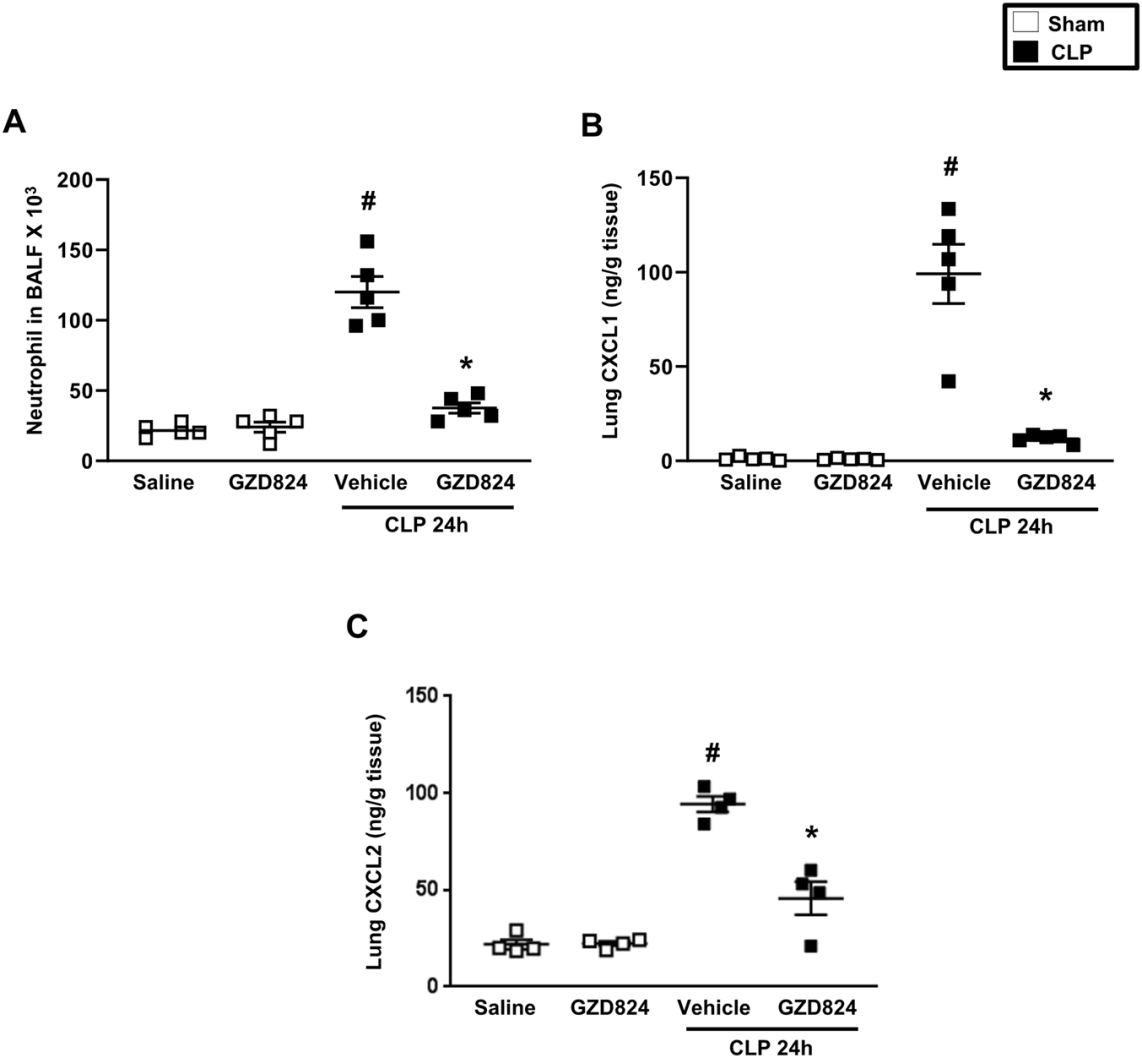
c-Abl kinase regulates CLP-induced infiltration of neutrophils in the lung. **A** Number of BALF neutrophils were determined 24 h after CLP induction. Pulmonary levels of **B** CXCL1 and **C** CXCL2. Animals were treated with GZD824 (5 mg/kg) or vehicle prior to CLP induction. Mice treated with saline (sham) or GZD824 alone without CLP. Mice treated with PBS served as sham animals. Data are presented as mean values ± standard error of the mean (SEM) and *n* = 5. ^#^*P* < 0.05 vs. Sham and *P < 0.05 vs. Vehicle + CLP.

The number of BALF neutrophils increased from 21.6 ± 4.5 × 10^3^ in sham mice to 120.0 ± 24.66 × 10^3^ in CLP mice, corresponding to a 6-fold increase (Fig. 6A). Blocking c-Abl kinase activity decreased the number of BALF neutrophils by 83% in septic mice (Fig. 6A). Moreover, CLP markedly increased pulmonary formation of CXCL1 and CXCL2 and treatment with GZD824 reduced CXC chemokine generation by more than 89% and 87% in CLP animals (Fig. 6B and C).

### c-Abl kinase regulates systemic inflammation in sepsis

**Fig. 7:**
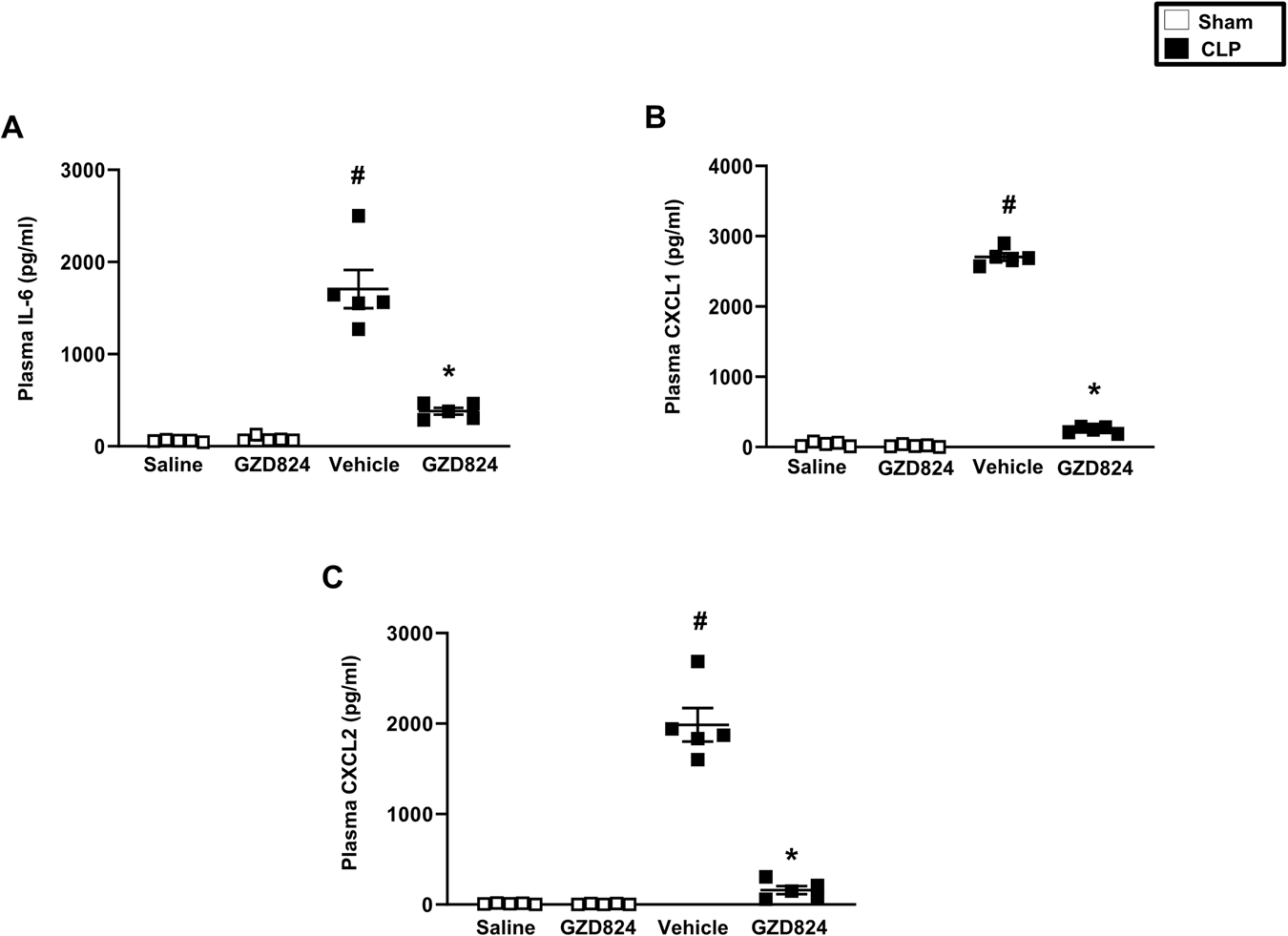
c-Abl kinase regulates CLP-induced systemic inflammation. Plasma levels of **A** IL-6, **B** CXCL1, and **C** CXCL2. Animals were treated with GZD824 (5 mg/kg) or vehicle prior to CLP induction. Mice treated with PBS (sham) or GZD824 alone without CLP. Data are presented as mean values ± standard error of the mean (SEM) and *n* = 5. ^#^*P* < 0.05 vs. Sham and *P < 0.05 vs. Vehicle + CLP.

Induction of CLP enhanced plasma levels of IL-6 by 29-fold, CXCL1 by 64-fold and CXCL2 by 37-fold (Fig. 7A–C). Administration of GZD824 decreased CLP-evoked plasma levels of IL-6, CXCL1 and CXCL2 by more than 80%, 92% and 92%, respectively (Fig. 7A–C). As part of a systemic inflammatory response in sepsis, the number of circulating leukocytes decreases. Indeed, it was observed that CLP caused leukocytopenia (Table 1) and that administration of GZD824 antagonized CLP-induced leukocytopenia in septic mice (Table 1).

**Table 1:** Systemic leukocyte differential counts.

	MNL	PMNL	Total
Sham + Saline	6 ± 0.2	1.5 ± 0.2	7.3 ± 0.6
Sham + GZD824	5.8 ± 0.4	1.3 ± 0.1	7.3 ± 0.5
CLP + Vehicle	1.1 ± 0.1*	0.4 ± 0.1*	2.8 ± 0.2*
CLP + GZD824	3.5 ± 0.4^†^	0.9 ± 0.1^†^	4.6 ± 0.7^†^

Blood was collected from sham animals receiving saline only as well as mice treated with GZD824 (5 mg/kg) or vehicle intravenously prior to cecal ligation and puncture for 24 h. Cells were identified as monomorphonuclear leukocytes (MNL) and polymorphonuclear leukocytes (PMNL). Data are presented as mean values ± standard error of the mean (SEM), 10^6^ cells/ml and *n* = 5. *P < 0.05 vs. Sham and ^†^*P* < 0.05 vs. Vehicle + CLP.

## Discussion

Our novel data suggest that c-Abl kinase plays a critical role in the development of abdominal sepsis. These results demonstrate that c-Abl kinase is a key regulator of NET formation in neutrophils and sepsis. In addition, it was found that targeting c-Abl kinase activity not only reduces NET generation but also decreases CLP-induced neutrophil infiltration and lung damage, suggesting that c-Abl kinase might be a useful target in abdominal sepsis.

Convincing data have documented that c-Abl kinase co-ordinate key aspects of actin dynamics and cytoskeletal rearrangements in cells^30,31^. Most knowledge about the function of c-Abl kinase has been obtained from studies of human leukemias^16,32^ but a growing body of evidence indicate that c-Abl also regulates critical components of inflammation, such endothelial cell integrity and neutrophil adhesion^22,23^. Moreover, the role of c-Abl kinase has also been implicated in inflammatory diseases, including immunoglobulin-mediated glomerular damage, nephrotoxicity and endotoxin-induced vascular leakage^20,21,33^. Herein, we found that induction of abdominal sepsis markedly increased c-Abl kinase activity in circulating neutrophils. Administration of GZD824, a specific c-Abl inhibitor, decreased activation of c-Abl kinase in circulating neutrophils in CLP mice, suggesting that GZD824 is an effective inhibitor of c-Abl kinase in vivo. Knowing that neutrophils play a key role in septic lung injury^2,34,35^, we next asked whether c-Abl kinase is involved in CLP-induced pulmonary damage. Indeed, we found that treatment with GZD824 markedly reduced lung edema in abdominal sepsis. These findings are in line with a previous study showing that c-Abl kinase mediates pulmonary vascular leakage and tissue damage triggered by endotoxin^18,20^. Moreover, our data suggest that c-Abl kinase activity regulates induction of septic lung injury, which is in line with a previous report showing that c-Abl kinase regulates sepsis-induced vascular leakage^19^. In this context, it is interesting to note that c-Abl kinase controls downstream activity of small GTPases of the Rho family, such as Rac1 and RhoA^36,37^, which are known to regulate lung injury in abdominal sepsis^15,38^. Thus, it might be speculated that part of the protective effect of inhibiting c-Abl kinase could be related to downstream inhibition of Rac1 or RhoA signaling. Although our data suggest that c-Abl kinase in neutrophils play an important role in septic lung damage, our results do not exclude that c-Abl kinase activity also in other cells are involved in septic lung damage. For example, it has been shown that c-Abl kinase in endothelial cells plays a key function in regulating vascular permeability induced by pro-inflammatory mediators^39^. In addition, it should be noted that a previous study showed that inhibition of c-Abl kinase attenuates endotoxin-provoked pulmonary inflammation but exacerbates ventilator-induced lung damage, suggesting that pro-inflammatory effects of c-Abl kinase could be context-dependent^40^.

It is generally held that neutrophil infiltration is a rate-limiting step in septic lung damage^2,5^. For example, depletion of neutrophils or blocking neutrophil recruitment have repeatable been shown to protect against lung damage in abdominal sepsis^34,41^. Herein, it was observed that CLP increased the number of neutrophils in the bronchoalveolar space. Treatment with GZD824 markedly attenuated number of alveolar neutrophils in the lung, suggesting that c-Abl kinase is a potent regulator of neutrophil recruitment in septic lung injury. Considering the important function of neutrophils in abdominal sepsis^2,3,5^, it could be forwarded that the inhibitory impact of GZD824 on neutrophil accumulation help to explain the protective effect of GZD824 in septic lung damage. Secretion of CXC chemokines, such as CXCL1 and CXCL2, co-ordinates neutrophil trafficking to sites of inflammation^42–45^. CLP triggered massive formation of CXCL1 and CXCL2 in the lung. Administration of GZD824 markedly reduced pulmonary formation of CXCL1 and CXCL2, suggesting that c-Abl kinase regulates CXC chemokine generation in the inflamed lung, which could help to explain part of the inhibitory impact of GZD824 on neutrophil recruitment in septic lung injury. Leukocytopenia is a hallmark of systemic inflammation^35^. In the present study, it was found that GZD824 antagonized CLP-induced leukocytopenia, indicating that c-Abl kinase also regulates systemic inflammation is sepsis. This notion is also supported by our results showing that GZD824 decreased plasma levels of IL-6, CXCL1, and CXCL2 in septic animals.

Published data in the literature suggest that NETs exert dual roles in infectious diseases. On one hand neutrophil-derived NETs protects against infections by trapping microbes and facilitate interactions between antimicrobial proteins and bacteria leading to microbiological clearance^12,46^. On the other hand, excessive NET generation causes tissue damage in both infectious and non-infectious diseases^10,47,48^. Numerous studies have documented that formation of neutrophil-derived NETs constitute a key component in the pathophysiology of sepsis^10,12,49^. In the present study, we observed that treatment with GZD824 reduced DNA structures co-localizing with the neutrophil-derived granule protein elastase and citrullinated histone 3 in the lung, indicating that c-Abl regulates NET formation in septic lung injury. Moreover, GZD824 decreased CLP-induced formation DNA-histone complexes in the plasma, supporting the notion above that c-Abl plays an important role in NET generation in abdominal sepsis. We then asked whether c-Abl kinase directly regulates NET formation in neutrophils. For this purpose, neutrophils were isolated and challenge with TNF-α, a cell wall component of *Saccharomyces cerevisiae* known to activate c-Abl kinase pathway, which significantly increased expression of MPO and citrullinated histone 3 on expelled neutrophil-derived DNA. Moreover, TNF-α stimulation of isolated neutrophils also enhanced formation DNA-histone complexes. We found that co-incubation with GZD824 decreased TNF-α-induced co-expression of MPO and citrullinated histone 3 on neutrophil-derived DNA as well as DNA-histone complexes in isolated neutrophils, indicating that c-Abl kinase directly controls NET formation in neutrophils. Together, these findings constitute the first evidence in the literature suggesting that c-Abl kinase regulates NET formation in neutrophils and sepsis. Notably, one study has reported that induction of ROS is pivotal in TNF-α-induced NET formation in neutrophils^50^. Herein, we found that inhibition of c-Abl kinase markedly reduced TNF-α-triggered formation of ROS in neutrophils. Thus, c-Abl kinase-dependent generation of ROS might be involved in TNF-α-induced formation of NETs in neutrophils. Although neutrophil-derived NETs are recognized for their important role in the innate immune system by trapping and killing microbes facilitating microbiological clearance^12,46^, several studies have shown that excessive formation of NETs is known to cause tissue injury and organ failure in infectious conditions^10,47^. Thus, pharmacological targeting of c-Abl kinase function might be a useful strategy to treat infectious diseases in which excessive NET formation harm host organs tissue while simultaneously antagonizing microbial pathogens. In this context, it should be noted that timing of anti-inflammatory intervention is critical in infectious diseases considering the important role of the immune system in bacterial clearance unless effective antibiotic coverage is provided throughout the treatment.

To conclude, these results show that c-Abl kinase plays a pivotal role in NET generation in neutrophils and in septic lung injury. Our findings indicate that targeting c-Abl kinase activity decreases pulmonary formation of CXC chemokines in sepsis. In addition, c-Abl kinase inhibition reduced neutrophil recruitment and tissue injury in lung. Finally, blocking c-Abl kinase activity decreased systemic inflammation and pulmonary neutrophilia in mice with sepsis. Thus, this study not only delineates a novel signaling mechanism regulating NET formation in sepsis but also suggests that blocking c-Abl kinase might be a useful strategy to ameliorate local and systemic inflammation in sepsis.

## Supplementary information

Supplemental figure 1
