# Supplementary figures and images for: c-Abl kinase regulates neutrophil extracellular trap formation and lung injury in abdominal sepsis
Source: Lab Invest. 2021 Nov 3;102(3):263–71. doi: 10.1038/s41374-021-00683-6 (PMC8860741; doi:10.1038/s41374-021-00683-6)

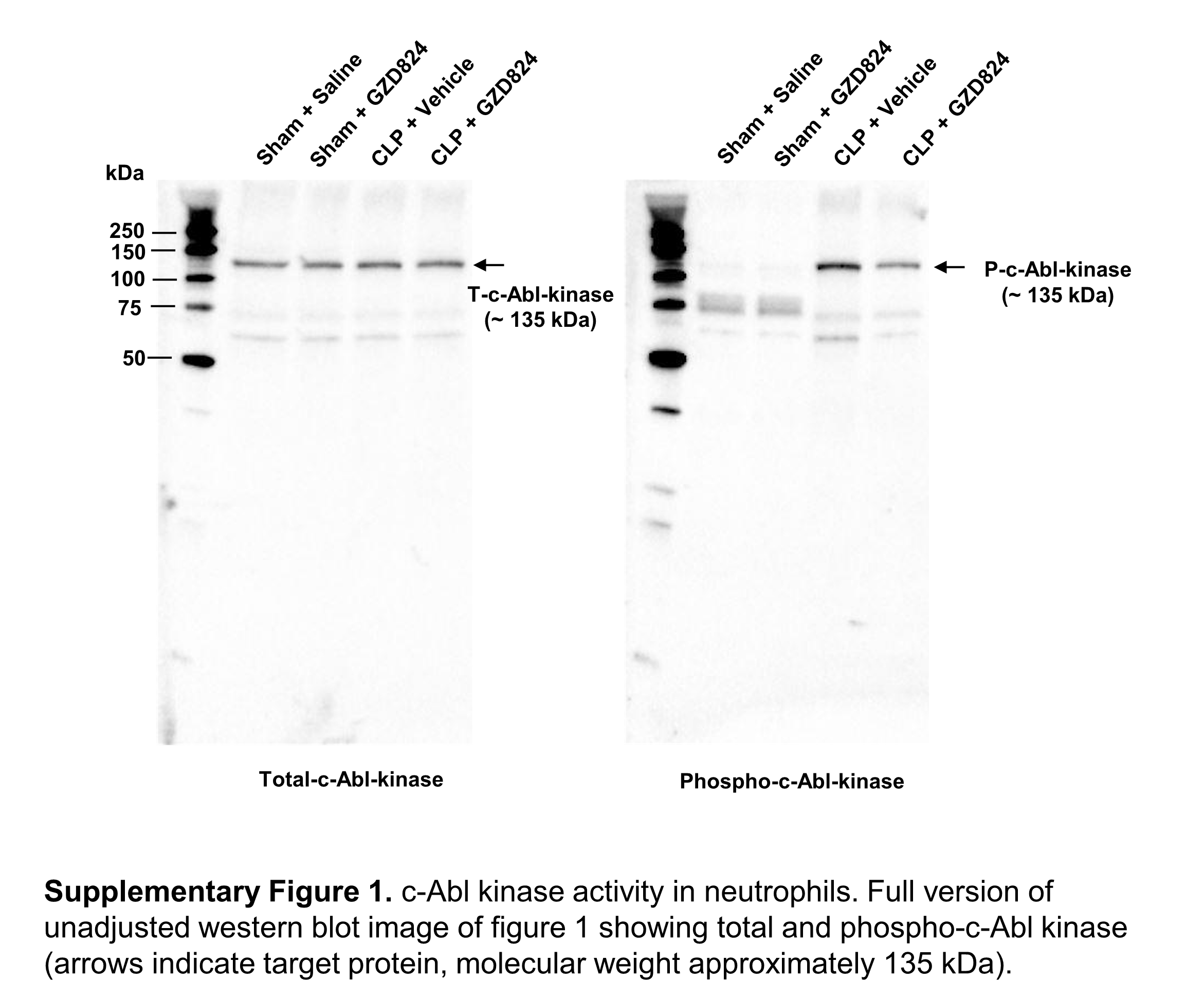

Supplement: Supplementary file 1 — Supplemental figure 1 [file 41374_2021_683_MOESM1_ESM.tif]
